# Intervals to *Plasmodium falciparum* recurrence after anti-malarial treatment in pregnancy: a longitudinal prospective cohort

**DOI:** 10.1186/s12936-015-0745-9

**Published:** 2015-05-28

**Authors:** Natthapon Laochan, Sophie G. Zaloumis, Mallika Imwong, Usa Lek-Uthai, Alan Brockman, Kanlaya Sriprawat, Jacher Wiladphaingern, Nicholas J. White, François Nosten, Rose McGready

**Affiliations:** Shoklo Malaria Research Unit, Mahidol-Oxford Tropical Medicine Research Unit, Faculty of Tropical Medicine, Mahidol University, Mae Sot, Thailand; Centre for Epidemiology and Biostatistics, Melbourne School of Population and Health, University of Melbourne, Melbourne, Australia; Mahidol-Oxford Tropical Medicine Research Unit (MORU), Faculty of Tropical Medicine, Mahidol University, Bangkok, Thailand; Centre for Tropical Medicine and Global Health, Nuffield Department of Medicine, University of Oxford, Oxford, UK; Department of Parasitology and Entomology, Faculty of Public Health, Mahidol University, Bangkok, Thailand

## Abstract

**Background:**

*Plasmodium falciparum* infections adversely affect pregnancy. Anti-malarial treatment failure is common. The objective of this study was to examine the duration of persistent parasite carriage following anti-malarial treatment in pregnancy.

**Methods:**

The data presented here are a collation from previous studies carried out since 1994 in the Shoklo Malaria Research Unit (SMRU) on the Thailand-Myanmar border and performed using the same unique methodology detailed in the Materials and Methods section. Screening for malaria by microscopy is a routine part of weekly antenatal care (ANC) visits and therapeutic responses to anti-malarials were assessed in *P. falciparum* malaria cases. Women with microscopy confirmed *P. falciparum* malaria had a PCR blood spot from a finger-prick sample collected. Parasite DNA was extracted from the blood-spot samples using saponin lysis/Chelex extraction method and genotyped using polymorphic segments of MSP1, MSP2 and GLURP. Recurrent infections were classified by genotyping as novel, recrudescent or indeterminate. Factors associated with time to microscopy-detected recrudescence were analysed using multivariable regression techniques.

**Results:**

From December 1994 to November 2009, 700 women were treated for *P. falciparum* and there were 909 recurrent episodes (481 novel and 428 recrudescent) confirmed by PCR genotyping. Most of the recurrences, 85 % (770/909), occurred after treatment with quinine monotherapy, artesunate monotherapy or artesunate-clindamycin. The geometric mean number of days to recurrence was significantly shorter in women with recrudescent infection, 24.5 (95 %: 23.4-25.8), compared to re-infection, 49.7 (95 %: 46.9-52.7), P <0.001. The proportion of recrudescent *P. falciparum* infections that occurred after days 28, 42 and 63 from the start of treatment was 29.1 % (124/428), 13.3 % (57/428) and 5.6 % (24/428). Recrudescent infections ≥100 days after treatment occurred with quinine and mefloquine monotherapy, and quinine + clindamycin and artesunate + atovaquone-proguanil combination therapy. Treatments containing an artemisinin derivative or an intercalated *Plasmodium vivax* infection increased the geometric mean interval to recrudescence by 1.28-fold (95 % CI: 1.09-1.51) and 2.19-fold (1.77-2.72), respectively. Intervals to recrudescence were decreased 0.83-fold (0.73-0.95) if treatment was not fully supervised (suggesting incomplete adherence) and 0.98-fold (0.96-0.99) for each doubling in baseline parasitaemia.

**Conclusions:**

Prolonged time to recrudescence may occur in pregnancy, regardless of anti-malarial treatment. Long intervals to recrudescence are more likely with the use of artemisinin-containing treatments and also observed with intercalated *P. vivax* infections treated with chloroquine. Accurate determination of drug efficacy in pregnancy requires longer duration of follow-up, preferably until delivery or day 63, whichever occurs last.

## Background

In order to reduce malaria-related maternal mortality in a low-transmission area where multidrug-resistant (MDR) strains of *Plasmodium falciparum* are prevalent, frequent screening and treatment of all positive malaria episodes is required. This has been a focus area of the antenatal clinics (ANCs) of Shoklo Malaria Research Unit (SMRU) on the Thailand-Myanmar border [1, 2]. In 1994, PCR genotyping of *P. falciparum* parasite recurrence was introduced to distinguish novel or new infections from recrudescent infections [3]. This is the accepted method for reporting trials of anti-malarial drug efficacy [4, 5]. PCR genotyping results of *P. falciparum* at SMRU have been published within the context of treatment efficacy trials in pregnant women [6–11] and in non-pregnant patients [12, 13]. The longest reported time to recrudescence confirmed by PCR genotyping in non-pregnant patients is 62 days [3] but the duration of follow-up in *P. falciparum* anti-malarial drug trials is limited to 63 days [4]. In pregnancy, when a woman may naturally follow antenatal care for an extended period, much longer carriage times have been reported: 133 days in Malawi [14], 187 days in Mozambique [15], 85 days [7], 98 days [10] and 121 days [3] on the Thailand-Myanmar border.

In the era of malaria therapy from the 1920s to the 1950s, and the volunteer studies conducted to assess new anti-malarial drugs conducted in Australia and the USA, natural infections in previously non-immune subjects were observed to last for many months. Transfusion malaria infections acquired from donors who had left the endemic area years previously were also documented [16]. In the early 1950s, Eyles and Young [17] reported the duration of incompletely treated, artificial *P. falciparum* infections (South Carolina strain) in neurosyphilis patients. The average duration of infection was 222 days with three of the infections persisting for more than one year, and the longest 480 days. In a similar report with the Panama strain of *P. falciparum* there were 23 incompletely treated infections which persisted for an average of 280 ± 20 (range 114 to 503) days, with four cases persisting for more than one year [18]. These reports occurred long before the PCR genotyping technique was introduced [19, 20] but the concept of prolonged asymptomatic carriage of *P. falciparum* is well established [21]. The longest recorded persistence of *P. falciparum* in an individual is 13 years [16]. Here *P. falciparum* genotyping was used to examine the duration of persistent *P. falciparum* carriage in pregnant women and examine the factors associated with time to recrudescence.

## Methods

### Study site and population

Pregnant women in this series attended the weekly ANC of SMRU on the northwestern border of Thailand. In this hilly, forested area malaria transmission is low and seasonal (estimated entomological inoculation rate <2) in the whole population, including pregnant women [22]. Acquired immunity is poorly protective and severe malaria is common at all ages, especially in pregnant women [22]. At the start of the research programme, maternal mortality from falciparum malaria was high: estimated 1,000 maternal deaths per 100,000 live births [2]. Women are invited to come to the ANC as soon as they are aware of their pregnancy. All women attending consultation are screened weekly for malaria as the only effective strategy to prevent maternal death [1]. Prophylactic vitamins (ferrous sulphate, folic acid and vitamin B1) are provided to all women attending for consultation. Haematocrit levels are measured at two weekly intervals and anaemic women (haematocrit <30 %) receive treatment doses of ferrous sulphate and folic acid. In the case of severe symptomatic anaemia (haematocrit <20 %) screened blood transfusions are provided.

### Treatment of *Plasmodium falciparum* in pregnancy

**Fig. 1:**
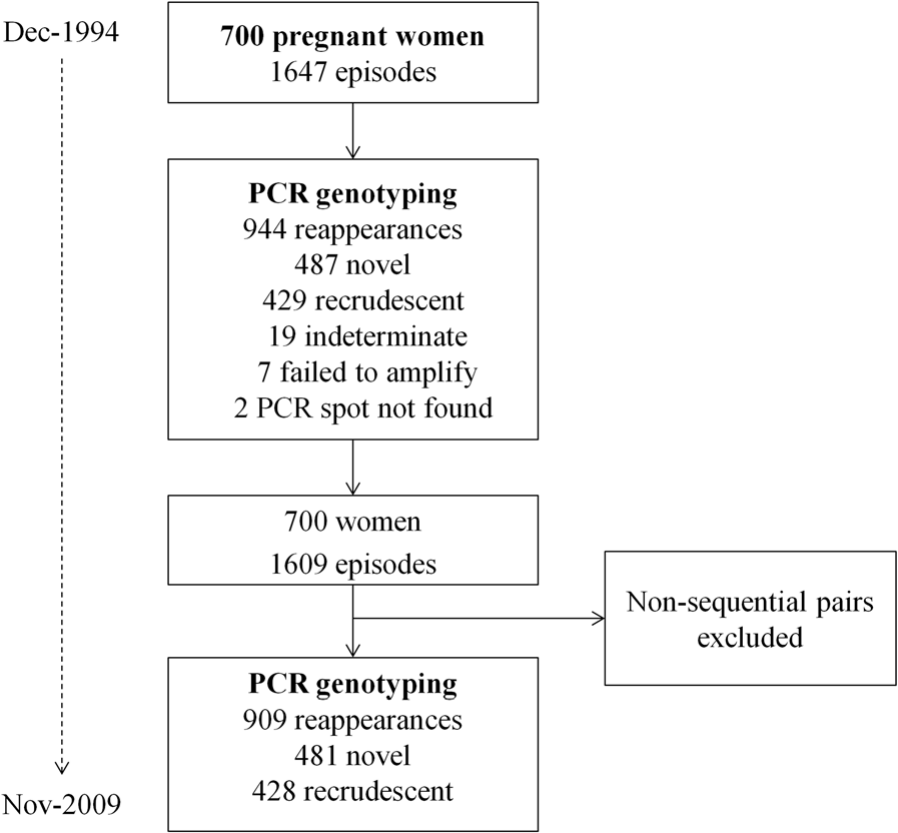
Study flow

This study spans 15 years during which treatment of falciparum malaria has changed from monotherapy to artemisinin-based combination therapy (ACT) (Fig. 1). Quinine based therapy has always been the first-line treatment for uncomplicated malaria in the first trimester. Quinine was also used throughout pregnancy until artemisinins were recommended in 2005 for routine first-line therapy in the second and third trimesters for uncomplicated malaria. Quinine-clindamycin (a non-artemisinin based combination therapy) has been used almost exclusively in the first trimester since its introduction in 2006.

Treatment doses for uncomplicated malaria infections were prescribed as follows: quinine three times daily (10 mg salt/kg every eight hours, Government Pharmaceutical Organization) for seven days; artesunate (outside the first trimester) once daily (usually 2 mg /kg) for seven days (total dose 10–16 mg/kg, Guilin, PRC); artemether-lumefantrine (artemether 20 mg and lumefantrine 120 mg, Novartis, Basel, Switzerland): four tablets twice daily for three days, mefloquine 25 mg/kg stat dose or as a split dose (15 mg/kg and 10 mg/kg on consecutive days); dihydroartemisinin-piperaquine once daily for three days (DHA 3 mg/kg, PPQ 15–17 mg/kg, Holley Pharm PRC); artesunate (4 mg/kg/day) combined to atovaquone-proguanil (atovaquone 20 mg/kg/day and 8 mg/kg/d) for three days (Malarone, Glaxo-Wellcome, Dartford, UK). Since 2007, clindamycin was combined with artesunate or quinine at a dose of 5 mg/kg three times daily (in practice in most women this was 300 mg three times daily, Siam Bheasach). Chloroquine (Government Pharmaceutical Organization, Thailand) was given once a day for three days, with a dose of 10 mg base/kg/day for two days and 5 mg base/kg/day on the third day, for *Plasmodium vivax.* Treatment administration was either unsupervised, when a pregnant woman was not admitted for treatment, or supervised when the initial doses or all of the doses were administered by a health worker and the pregnant woman was observed for vomiting. Pregnant women who vomited within 30 min of their dose were re-administered a full dose and those who vomited within 30 to 60 min of initial dosing were re-administered half the dose.

Contrary to the changes in treatment over time there has been no change in the unique methodology applied to pregnant women in this area including the screening by microscopy for malaria offered at every antenatal visit during pregnancy, in the basic data collected at the time of each malaria episode and in the way the sample is collected. At the time of each malaria episode the date, body temperature, history of fever in the previous 48 h, parasitaemia, haematocrit, gestational age of the pregnancy, and malaria history were recorded. After microscopy diagnosis and before treatment, fresh blood from a finger-prick sample was used to make three blood spots (approximately 30 μl each) on a strip of 3 M filter paper. This was dried overnight before DNA extraction the next day.

### Definitions

Symptomatic malaria was defined as slide-confirmed parasitaemia with a history of fever in the previous 48 h or a measured axillary or aural temperature ≥37.5 °C [23]. Asymptomatic malaria was defined as slide-confirmed parasitaemia without any history of fever and a measured temperature <37.5 °C. The gestational age at the time of the malaria episode was determined by ultrasound [24], the Dubowitz newborn examination [25] or by the fundal height formula developed for this population [26]. Treatments were classified as monotherapy when given without any other anti-malarial. Mefloquine and piperaquine were categorized as ‘long-acting’ drugs, lumefantrine and atovaquone-proguanil as medium-acting drugs and the remainder as short acting.

For recrudescence risk factor based analysis anti-malarial therapies were grouped into artemisinin based: including ACT and artesunate monotherapy and non-artemisinin based including combinations such as quinine and clindamycin or monotherapy such as mefloquine.

### *Plasmodium falciparum* PCR genotyping

*Plasmodium falciparum* infection from consecutive malaria episodes during pregnancy was analysed by using the three loci genotypes: MSP1, MSP2 and GLURP. Nested PCR was the amplification strategy used to genotype *P. falciparum* and this is explained in detail elsewhere [3, 27, 28]. A parasite infection with the same three-locus genotype pre- and post-treatment was considered a recrudescence. Infections which differed pre- and post-treatment were denoted as novel (or new) infections. Infections that could not be classified as recrudescent or new were denoted as indeterminate. Only patients who became microscopy smear negative following treatment were included in the analysis. The time to the next patent infection was calculated by the difference in days from the date treatment started until the date of the first recurrence detected by microscopy with active weekly screening.

### Statistical analysis

**Fig. 2:**
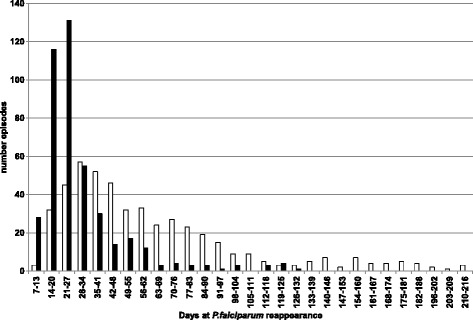
Frequency of PCR-confirmed novel (*white*) and recrudescent (*black*) *Plasmodium falciparum* infections for each follow-up week from day 7 of treatment

Continuous variables were described using median (range) and categorical variables using frequency (percentage). The distribution of the number of days to recurrence of *P. falciparum* infection was positively skewed (Fig. 2) and therefore natural logarithm (log_e_) transformed. Linear regression models were fitted to the log_e_ transformed outcome, and either geometric means or ratios of geometric means were derived. Separate sub-group analyses were performed for women with recrudescent infection(s) and women with novel infection(s). In the multivariable linear regression analyses, a risk factor was deemed to be associated with the log_e_ transformed outcome if the p-value was below the nominal significance level of 0.1; multi-collinearity between each of the covariates (i.e., risk factors) was assessed using variance inflation factors (VIFs; all <2); and all estimates derived from the regression analyses were adjusted for age (years), weight (kg), and study period categorized into four three-year intervals (1994–97, 1998–2001, 2002–05, 2006–09). As resistance appears with increasing years of drug use, e.g., artemisinin, and as the cohort was conducted over many years with different numbers of women available in different years, four blocks were chosen for a reasonably even distribution of women in each block, so time could be controlled for in regression analysis. To account for the correlation between the number of days to recurrence of multiple *P. falciparum* infections during a single pregnancy, robust standard errors were calculated using the Huber-White sandwich estimator. Tests for linear trends were performed by fitting a linear model to the outcome variable with the categorical covariate treated as pseudo continuous. Proportions were compared using Pearson’s Chi-squared test. Data were analysed in Stata/IC version 11.1 (StataCorp, College Station, Texas, USA).

### Ethics approval

The data presented here represent a collation of data from women enrolled in treatment or in one prevention trial in pregnancy all previously published and approved by the Faculty of Tropical Medicine Ethics Committee in Bangkok and in later years the Oxford Tropical Research Ethics Committee [6–11, 29].

## Results

From December 1994 to November 2009, there were 700 women with 1,647 episodes of *P. falciparum* of which 19 were classified as indeterminate by PCR; seven failed to amplify; no PCR spot was found for two episodes and pregnancies with episodes of non-sequential pairs were removed, leaving 1,609 episodes for analysis in 700 women (Fig. 1). Most of the women, 691 (76.0 %), experienced a single recurrence of *P. falciparum* during their pregnancy, 164 (18.0 %) had two and 54 (6.0 %) experienced three or more recurrences within the same pregnancy. The maximum number of consecutive *P. falciparum* episodes within a single pregnancy was five and all four recurrent infections could be characterized by PCR in two women. The demographic characteristics of the pregnant women at the time of the primary infection are reported in Table 1.

**Table 1:** Distribution of variables measured in 700 pregnant women before treatment of the primary *Plasmodium falciparum* infection

Characteristic	N^a^	Median [range] or %
Age (years)	700	24.5 [14–45]
Maternal weight (kg)	692	47 [30–78]
Haematocrit (%)	660	31 [12–48]
Parasitaemia/μL	699	3,216 [16.4-4,768]
Temperature (°C)	642	36.8 [35.0-40.4]
Symptomatic	654	67.3

^**a**^N, number of observations

Most of the recurrent infections, 85 % (770/909), occurred following treatment with one of the three most commonly used regimens: quinine monotherapy, artesunate monotherapy or artesunate-clindamycin, which are all rapidly eliminated seven-day treatments (Table 2). Not all drugs were used for treatment across all years of the data collection period, for example, mefloquine monotherapy was only administered from 1994 to 1997 and in a very small number of women (Table 2). The mean gestational age at the time of treatment was lowest for quinine as it is the drug of choice for first trimester (Table 2). The number of recurrent infections classified as recrudescent or novel varied with the anti-malarial treatment provided (Table 2).

**Table 2:** Estimated gestational age and time to recurrence of *Plasmodium falciparum* (*Pf*) for novel and recrudescent infections for each anti-malarial treatment and including the distribution of intercalated *Plasmodium vivax* infections

		EGA^d^ (weeks)	Time to novel infection (days)		Time to recrudescent infection (days)					Intercalated *P. vivax*
Anti-malarial (action, years)^a^	N^b^	Median [range]	N	Median [range]	N	Median [range]	% >28 days	% >42 days	% >63 days	% (N)^c^
Quinine monotherapy (short, 1994–2006)	476	17 [3–36]	173	47 [7–216]	303	21 [7–126]	25.4 (77/303)	10.6 (32/303)	5.0 (15/303)	33.0 (58)
Quinine + clindamycin (short, 1998–2009)	29	10 [4–24]	22	84 [28–201]	7	50 [21–122]	57.1 (4/7)	57.1 (4/7)	42.9 (3/7)	7.4 (13)
Artesunate monotherapy (short, 1995–2008)	151	21 [4–37]	102	54 [8–182]	49	22 [11–99]	30.6 (15/49)	12.2 (6/49)	4.1 (2/49)	21.6 (38)
Artesunate + Clindamycin (short, 1998–2009)	143	23^e^ [1–39]	121	49 [21–158]	22	28 [12–70]	45.5 (10/22)	27.3 (6/22)	9.1 (2/22)	26.1 (46)
Artemether + lumefantrine (medium, 2004–2006)	43	23 [13–36]	24	36 [15–140]	19	23 [14–63]	31.6 (6/19)	26.3 (5/19)	0.0 (0/19)	6.3 (11)
Artesunate + Atovaquone + proguanil (medium, 1999–2005)	11	23 [18–32]	8	67 [44–101]	3	49 [35–120]	100.0 (3/3)	66.7 (2/3)	33.3 (1/3)	1.7 (3)
Mefloquine monotherapy (long, 1994–1997)	22	26 [12–38]	4	25 [9–44]	18	22 [7–123]	27.8 (5/18)	5.6 (1/18)	5.6 (1/18)	0 (0)
DHA^f^ + Piperaquine (long, 2006–2008)	29	20 [8–34]	23	63 [35–180]	6	33 [21–49]	66.7 (4/6)	16.7 (1/6)	0.0 (0/6)	4.0 (7)
Artesunate + Mefloquine (long, 1995–2008)	5	28 [−0.4-39]	4	35 [20–43]	1	21 [−]	0.0 (0/1)	0.0 (0/1)	0.0 (0/1)	0 (0)

^a^action, reflects length of time anti-malarial remains in the body after administration; years, period in which treatment was administered at SMRU

^b^N, number of recurrent *Pf* episodes

^c^% (N), percentage and number of intercalated *P. vivax* episodes

^d^EGA, estimated gestational age (weeks) at time of malaria episode

^e^one EGA missing

^f^DHA, dihydroartemisinin

### Time to recurrence in recrudescent and novel infections

The geometric mean number of days to recurrence was significantly shorter in women with recrudescent infection, 24.5 (95 %: 23.4-25.8) days, compared to re-infection, 49.7 (95 %: 46.9-52.7) days, *P* <0.001 (Fig. 2). There was no statistically significant difference in the geometric mean number of days to recurrence according to the episode number, i.e., for recurrent episodes that were recrudescent infections, the time from the primary infection to first recrudescence (*N* = 353) was 24 (95 % CI: 23–26) days; first to the second recrudescence (*N* = 55) was 25 (95 % CI: 22–29) days; and, from the second to the third or more recrudescence (*N* = 20) was 28 (95 % CI: 22–35) days, *P* = 0.44. For recurrent episodes that were novel infections, the observed times were 50 (95 % CI: 46–53) days for primary to first (*N* = 339); 51 (95 % CI: 46–58) days first to second (*N* = 108); and, 45 (95 % CI: 38–53) days for second to third or more infection (*N* = 34), *P* = 0.38.

### Time to recurrence with intercalated *Plasmodium vivax* infection

Of the 909 *P. falciparum* recurrences, 19.3 % (176) had an intercalated *Plasmodium vivax* infection between the *P. falciparum* primary and recurrent episode. There were 145 recurrent *P. falciparum* episodes with one intercalated *P. vivax* episode; and 23, seven and one with recurrent *P. falciparum* episodes with two, three and five intercalated *P. vivax* episodes, respectively. The proportion of recrudescent *P. falciparum* infections following an intercalated *P. vivax* infection was significantly lower than those without an intercalated *P. vivax* episode: 19.9 % (35/176) compared with 53.6 % (393/733), P <0.001. In both novel and recrudescent infection, intercalated *P. vivax* infection resulted in a longer geometric mean number of days to recurrence compared to those without intercalated *P. vivax*: 71 (95 % CI: 65–78) versus 43 (95 % CI: 40–46) days, and 49 (95 % CI: 41–59) versus 23 (95 % CI: 22–24) (P <0.001 for both), respectively. The proportion of intercalated *P. vivax* infections was lower with medium- and long-acting anti-malarial treatments (Table 2).

### Time to recurrence according to anti-malarial treatment

The geometric mean number of days to recrudescent infection(s) for short- (*N* = 381), medium- (*N* = 22) and long- (*N* = 7) acting anti-malarials was 24 (95 % CI: 23–26), 28 (95 % CI: 25–33) and 33 (95 % CI: 26–43) days (linear trend: *P* = 0.02), when mefloquine monotherapy was excluded from the analysis (only used from 1994–97, but high grade resistance was already established during this period). When mefloquine monotherapy was included, the geometric mean number of days to recrudescent infection for long-acting anti-malarials fell to 25 (95 % CI: 20–32) days and the linear trend was no longer significant (linear trend: *P* = 0.85). There was no evidence of a linear trend in number of days to novel infection for short-, medium- and long-acting anti-malarials.

### Recrudescence after 28, 42 and 63 days of follow-up in pregnancy

Nearly one third 29 % (124/428) of recrudescent infection occurred after day 28 with 13.3 % (57/428) and 5.6 % (24/428) occurring after day 42 and 63, respectively. The proportion of quinine treatments that were true recrudescent infections that would be wrongly classified as ‘treatment success’, if follow-up ceased at day 28, day 42 and day 63 were 26.1 % (81/310), 11.6 % (36/310) and 5.8 % (18/310), respectively (Table 2). For artemisinin-based treatments the respective proportions were 38.0 % (38/100), 20.0 % (20/100) and 5 % (5/100) (Table 2).

### Factors associated with time to a recrudescent infection

For four anti-malarial treatments (quinine and mefloquine monotherapy, quinine + clindamycin and artesunate + atovaquone + proguanil), women were observed to have recrudescent infections 100 or more days from the start of drug treatment (Table 2). There were no intercalated *P. falciparum* recrudescent infections (i.e., a recrudescence of a primary infection after an intervening novel infection).

Estimated ratios of geometric means are presented in Table 3 for eight risk factors hypothesized to be associated with the number of days to recrudescent *P. falciparum* infection. Of the eight risk factors, anti-malarial therapy, therapy supervision, intercalated *P. vivax* infection, and initial parasitaemia were found to be associated with number of days to recrudescent infection P <0.1, in linear regression analyses, including a single risk factor and adjusting for age, weight and study period (see Table 3 column ‘Univariable’). These variables remained associated at the nominal level of significance of 0.1, in a linear regression analysis including all risk factors and adjusting for age, weight and study period (see Table 3 column ‘Multivariable’). The estimated ratio of geometric means for the statistically, significantly associated, risk factors obtained from the multivariable analysis suggests an increase in the geometric mean number of days to recrudescent infection of 1.28-fold (95 % CI: 1.09-1.51) for women treated with artemisinin based therapy compared to non-artemisinin based therapy; and 2.19-fold (95 % CI: 1.77-2.72) in those women with an intercalated *P. vivax* infection compared to those who did not have intercalated *P. vivax*. A decrease in the geometric mean number of days to recrudescent infection of 0.83-fold (95 % CI: 0.73-0.95) was observed for women whose treatment was incompletely supervised compared to completely supervised; and 0.98-fold (95 % CI: 0.96-0.99) for each doubling in the baseline parasitaemia.

**Table 3:** The estimated ratio of the geometric mean number of days to recrudescence for each risk factor derived from linear regression analyses of 428 *Plasmodium falciparum* episodes among 354 pregnant women

		Univariable^a^		Multivariable^a^	
Risk factor	Categories	N^b^	Ratio of geometric means (95 % CI) [P-value]	N^b^	Ratio of geometric means (95 % CI) [P-value]
Parity	Primiparous	93	0.99 (0.86, 1.14) [0.890]	79	1.03 (0.89, 1.19) [0.698]
	Multiparous	256	1^c^	212	1
Anti-malarial therapy	Artemisinin based	49	1.28 (1.09, 1.51) [0.003]	38	1.24 (1.07, 1.43) [0.003]
	Non-artemisinin based	300	1	253	1
Symptoms	Symptomatic	232	0.89 (0.77, 1.03) [0.120]	211	0.94 (0.80, 1.11) [0.459]
	Asymptomatic	88	1	80	1
Therapy supervision	Unsupervised	86	0.83 (0.73, 0.94) [0.004]	81	0.83 (0.73, 0.95) [0.006]
	Supervised	253	1	210	1
Intercalated *P. vivax*	Yes	28	2.19 (1.77, 2.72) [<0.001]	25	2.19 (1.76, 2.73) [<0.001]
	No	321	1	266	1
EGA (weeks)	349	349	1.00 (0.99, 1.00) [0.217]	291	1.00 (0.99, 1.00) [0.210]
Parasitaemia (m/L)^d^	349	349	0.98 (0.97, 1.00) [0.079]	291	0.98 (0.96, 0.99) [0.014]
Haematocrit (%)	327	327	1.00 (0.99, 1.01) [0.618]	291	1.00 (0.99, 1.01) [0.654]

Combo, combination; Mono, monotherapy

^a^Outcome is time to recrudescent infection and estimates are adjusted for age, weight and study period. Univariable, linear model included a single risk factor, age, weight and study period; Multivariable, linear model included all risk factors, age, weight and study period

^b^The total number of episodes (N) included in each analysis may differ from 428 due to missing risk factor data

^c^Indicates reference group

^d^Parasitaemia was log_2_ transformed. Estimate interpreted as a 0.98-fold decrease in geometric mean time to recrudescent *Pf* infection(s) for a doubling in parasitaemia

## Discussion

This study reports the largest, single-site, longitudinal population study of PCR-genotyped *P. falciparum* malaria parasitaemia in pregnancy.

### Prolonged carriage in pregnant women

The interval from treatment of falciparum malaria in pregnancy to PCR-confirmed recrudescent infection can be prolonged. The maximum observed time in this large dataset was 126 days. Pregnant women in this study generally received supervised rather than unsupervised treatment of *P. falciparum*. Supervising the treatment was associated with an increased interval to late recrudescence. Several factors contributed to the high rates of recrudescence. While all women were treated with standard adult dosing of anti-malarials, pregnancy alters the pharmacokinetic properties of many anti-malarials lowering their plasma concentrations [17, 18]. It has been hypothesized that artesunate-treated parasites may enter a state of quiescence, which protects them from the lethal effects of anti-malarials but later allows recovery and normal growth [30, 31]. Dormancy has also been postulated to occur with pyrimethamine, atovaquone and proguanil [32, 33]. However, in this cohort, combination therapy whether artesunate-based or not was associated with a longer time to recrudescent infection. Immunity to malaria is reduced in pregnancy and the placenta provides a favourable site for the parasite to sequester where host defence mechanisms are attenuated [34].

Intercalated *P. vivax* infection resulted in a significantly lower proportion of recrudescent *P. falciparum* episodes than in women who did not have intercalated *P. vivax*. Intercalated *P. vivax* prolonged the time to recurrence of both novel and recrudescent infections and was the most significant risk factor for prolonged time to recrudescence. Although the chloroquine inhibitory concentration of 50 % (IC50) for *P. falciparum* isolates in this area are amongst the highest reported in the world [35, 36] some isolates are still partially sensitive to chloroquine. This is consistent with results that demonstrated a non-significant reduction in *P. falciparum* in a double-blind, randomized, placebo-controlled, prophylaxis trial of 1,000 pregnant women in this setting [29]. In addition, *P. vivax* may activate host-defences which affect *P. falciparum* [37]. The two parasites are competitive and provide mutual suppression. A protective effect of *P. vivax* has previously been reported in this area: mixed (*P. falciparum* and *P. vivax*) infection was associated with a four-fold reduction in the risk of developing severe malaria [22].

### Pregnancy and anti-malarial efficacy

The proportion of recrudescent infections with quinine and mefloquine monotherapy was high and these poorly efficacious regimens should no longer be used in this area [6, 7]. Patent and sub-patent *P. falciparum* parasitaemia in pregnancy are harmful to the pregnant woman and the foetus and prompt and efficacious treatment is required [38]. Frequent screening of a woman during pregnancy will increase the detection of parasitaemia [2]. Treatment of these episodes reduces but does not eliminate the adverse effects of low birth weight for the foetus and anaemia for the mother [2], which can only be achieved by prevention.

This study demonstrates that a significant proportion of pregnant women, 29.0 % by day 28, 13.3 % by day 42 and 5.6 % by day 63 of follow-up, have recrudescent infections beyond the traditional boundaries used for follow-up in anti-malarial efficacy trials. These proportions are large enough to underestimate significantly true failure rates with shorter periods of follow-up. Accurate assessment of anti-malarial efficacy in pregnancy requires follow-up to delivery, failing this, day 63. This is contrary to modelling data from trials in non-pregnant patients where 42–63 days follow-up captures nearly all anti-malarial treatment failures [4].

There are limitations to this analysis: only three drug treatments (quinine monotherapy, artesunate monotherapy and artesunate clindamycin) accounted for 85 % of the data. In this setting, with MDR-*P. falciparum* no trial that includes both pregnant and non-pregnant women has been conducted. Attempts to pool data on recrudescent *P. falciparum* infections for age-matched women of reproductive age treated with the same anti-malarial and same year of treatment within the population, i.e., artemether-lumefantrine and dihydroartemisinin-piperaquine, were unsuccessful.

While there are reasons to suspect that pregnancy *per se* many predispose to a longer duration to recrudescence this study is unable to prove it. Only settings with data including prolonged duration of follow-up; the same regimen for diagnosis and treatment of *P. falciparum* infections in pregnant and non-pregnant women which is most often not the case; and preferably with women matched for age and gravidity would have the potential to elucidate this suspicion.

### Post-treatment prophylactic effect

No effect of anti-malarial drugs was observed on the post-treatment prophylactic effect, i.e., the time to novel recurrence however without a group who receives no anti-malarials this cannot be proven. There was limited power to detect a post-treatment prophylactic effect and there were few patients who received long-acting anti-malarials and re-infection rates were very low, so any effect would have been small. Regarding IPT in this area a study conducted in men followed for nine months in a randomized, placebo controlled IPT trial required dihydroartemisinin-piperaquine to be provided monthly at full treatment doses (two tablets twice per day for three days) to be effective [39]. When re-infection rates of *P. falciparum* are very low it is difficult to justify the use of IPT as the risk benefit ratio may not be in favour of providing this drug to all pregnant women. The current annual incidence of *P. falciparum* in pregnancy in the area is now less than 0.5 infections per woman per year [40].

## Conclusion

The interval to recrudescence of falciparum malaria in pregnancy can be prolonged, regardless of the anti-malarial used for treatment. In areas where intercalated *P. vivax* occurs and receives treatment, time to recrudescence of *P. falciparum* can be prolonged. In this area of low, seasonal malaria transmission, recrudescence occurred after day 42 in approximately 15 % and after day 63 in 5 %, of pregnant women. Accurate characterization of drug efficacy in pregnancy requires follow-up to delivery or day 63, whichever occurs last.
